# Systematic literature review and clinical validation of circulating microRNAs as diagnostic biomarkers for colorectal cancer

**DOI:** 10.18632/oncotarget.19344

**Published:** 2017-07-18

**Authors:** Cheng Pan, Xuebing Yan, Hao Li, Linsheng Huang, Mingming Yin, Yongzhi Yang, Renyuan Gao, Leiming Hong, Yanlei Ma, Chenzhang Shi, Huanlong Qin, Peng Zhang

**Affiliations:** ^1^ Department of General Surgery, Shanghai Tenth People's Hospital Affiliated to Tongji University No. 301, Shanghai 200072, China; ^2^ Medical Department, Soochow University, Jiangsu 215123, China; ^3^ Department of General Surgery, Weihai Municipal Hospital, Shandong 264200, China

**Keywords:** colorectal cancer, circulating microRNAs, diagnosis, biomarkers

## Abstract

Because patients with colorectal cancer (CRC) are usually diagnosed at an advanced stage and current serum tumor markers have limited diagnostic efficacy, there is an urgent need to identify reliable diagnostic biomarkers. To better define the diagnostic potential of microRNAs (miRNAs) for CRC, we performed a comprehensive evaluation of reported circulating CRC miRNA markers. After a systematic literature review, we selected 30 candidate miRNAs and used quantitative real-time polymerase chain reaction to examine their expression in a training cohort of 120 plasma samples (CRC vs healthy controls (HC) = 60:60). Expression data was confirmed in a validation cohort of 160 plasma samples (CRC vs HC = 80:80). We ultimately identified 5 dysregulated circulating miRNAs (miR-15b, miR-17, miR-21, miR-26b, and miR-145), of which miR-21 and miR-26b proved to have the best diagnostic performance in the training and validation cohorts, respectively. Based on these results, we propose a novel blood-based diagnostic model, integrating 5 CRC-related miRNAs and serum carcinoembryonic antigen (CEA), which provides better diagnostic performance than the combined 5 miRNAs, CEA alone, or any single miRNA. We propose that the novel CRC diagnostic model presented here will be useful for overcoming the limitations faced by current non-invasive diagnostic strategies.

## INTRODUCTION

Colorectal cancer (CRC) is a commonly diagnosed cancer worldwide, and its incidence is dramatically increasing in developing countries with growing aging population and westernized lifestyles. Although population-based screening has proved to effectively prevent CRC development when detected at early stage, approximately 60% of CRC patients are diagnosed at regional or distant stages, with a discouraging 5-year survival rate ranging from 12.5% to 70.4% [1]. Moreover, when assessed alone, current tumor markers such as the carcinoembryonic antigen (CEA) and CA125 are frequently ineffective for early CRC detection, inevitably resulting in delayed diagnosis [2]. As numerous molecular biomarkers of CRC progression and prognosis have been recently proposed [3], there is substantial hope in combining them with conventional clinical parameters to more accurately diagnose CRC and guide treatment.

Several studies in the last few years have closely linked microRNAs (miRNAs) with the initiation and development of various human malignancies [4]. Several studies from our research group have also assessed the biological roles and relevant mechanisms of diverse miRNAs in CRC tumorigenesis. For example, we identified miR-17 as an oncogenic miRNA that promotes CRC development by activating the Wnt/β-catenin pathway by targeting P130 [5]. We also found that miR-149 methylation contributes to CRC growth and invasion by targeting the transcription factor Sp1 [6]. Furthermore, using miRNA expression profiling, we identified miR-150 as a prognostic biomarker for chemotherapy response and defined its anti-cancer effects, exerted through c-myb downregulation [7, 8]. Lastly, we defined a novel oncogenic role for miR-21 in the malignant transformation of colitis-associated CRC, where it targets the tumor suppressor PDCD4, activating the pro-inflammatory NF-kB/STAT3 cascade [9].

Given the crucial roles played by miRNAs in CRC development, there is great potential in translating them into clinically actionable biomarkers for diagnosis and prognostication. Studies revealed that tumor-derived miRNAs can be released into the circulation by exosomes, microvesicles, or bound to RNA binding proteins and lipoproteins [10, 11]. In 2009 Ng et al. identified for the first time a plasma miRNA marker, miR-92, that distinguished CRC patients from healthy controls with a sensitivity of 89% and a specificity of 70% [12]. Following his work, numerous studies reported the diagnostic value of other circulating miRNAs, such miR-21 and miR-221, in CRC patients [13–15]. However, these achievements have not been satisfactorily translated into clinical benefits largely due to insufficient retrospective validation on a highly standardized platform. Consequently, the clinical utility of combined miRNAs and their potential cooperation with traditional noninvasive diagnostic tumor markers (such as CEA) remain undetermined.

To address this issue, we performed a systematic literature review and selected candidate circulating miRNAs from relevant studies, including our own published work. Then, we employed a training cohort and a validation cohort to evaluate their diagnostic value. Finally, we constructed and validated a novel diagnostic model integrating multiple miRNAs and conventional tumor markers. These efforts not only provide a comprehensive evaluation of the diagnostic value of circulating miRNAs in CRC, but also strongly promote the clinical translation of a novel, non-invasive diagnostic approach.

## RESULTS

### Selection of candidate circulating miRNAs

**Figure 1:**
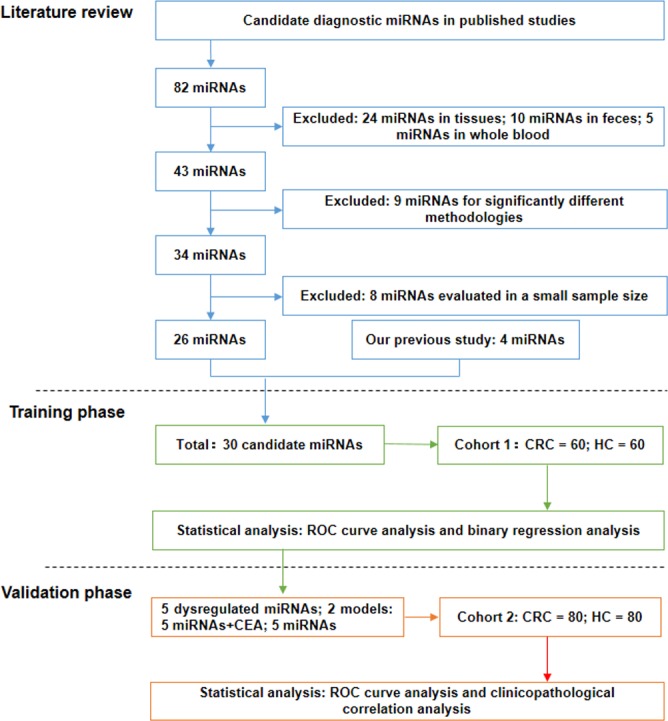
Study design flow chart The study consisted of three sections: a systematic literature review to select candidate miRNAs, a training phase for screening candidate miRNAs and constructing diagnostic models, and a validation phase for confirming optimal miRNAs/diagnostic models.

We initially performed a systematic literature review and selected 82 miRNAs with diagnostic potential that were detected in CRC patients (Figure 1). To further narrow our candidate list, we first excluded miRNAs detected in tissues (n = 24) and feces (n = 10). We also excluded miRNAs detected in whole blood (n = 5) because blood cells are a major contributor to circulating miRNA and may have an equivocal impact on analysis [16, 17]. We then excluded controversial miRNAs which were investigated by divergent methodologies (n = 9) and studies which enrolled fewer than 50 samples (n = 8). As a result, 26 miRNAs were preserved (Supplementary Table 1) [12, 14, 15, 18–39]. We added to this dataset 4 miRNAs (miR-17, miR-26b, miR-149, and miR-150) found to be involved in CRC development in our previous studies [5–7, 40]. Thus, a total of 30 miRNAs were finally selected for screening in the following training phase.

### Circulating miRNA screening and diagnostic model construction in the training phase

**Figure 2:**
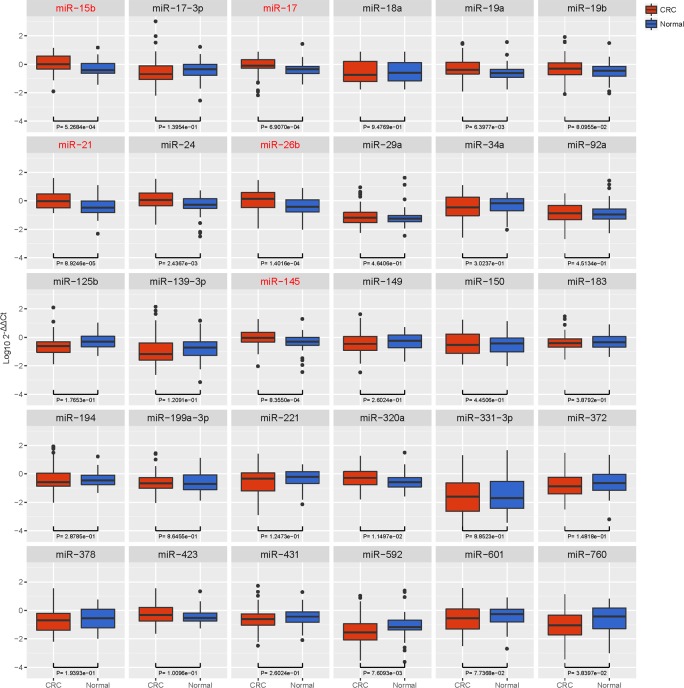
Box plot of the relative expression levels of candidate miRNAs in the training cohort (n = 120) The y axis indicates log_10_ 2^−△△Ct^ relative expression of miRNA. Among all the circulating miRNAs selected (n = 30), the expression of 5 miRNAs was significantly higher in CRC than in HC (miR-15b, p = 0.0005; miR-17, p = 0.0007; miR-21, p < 0.0001; miR-26b, p = 0.0001; miR-145, p = 0.0008).

In the training phase, qRT-PCR was first performed to compare the expression of candidate circulating miRNAs between CRC patients (n = 60) and healthy controls (HC) (n = 60) (Table 1). As shown in Figure 2, the expression of 5 circulating miRNAs was significantly higher in CRC than in HC (miR-15b, p = 0.0005; miR-17, p = 0.0007; miR-21, p < 0.0001; miR-26b, p = 0.0001; miR-145, p = 0.0008). No significant differences were observed between CRC and HC in the other 25 circulating miRNAs [p > 0. 0.0017 (0.05/30)].

**Table 1:** Clinicopathological characteristics of all the participants in the training and validation cohort

Characteristics	Training cohort		Validation cohort	
	CRC cases N=60	Healthy controls N=60	CRC cases N=80	Healthy controls N=80
Age(Mean±SD,years)	61.20±11.00	60.98±5.26	63.75±12.34	62.25±8.24
Gender				
Male	36 (60%)	31 (51.7%)	50 (62.5%)	47 (58.75%)
Female	24 (40%)	29 (48.3%)	30 (37.5%)	33 (41.25%)
T status				
T1	7	-	4	-
T2	5	-	5	-
T3	7	-	7	-
T4	41	-	59	-
Unknown	0		5	
N status				
N0	29	-	35	-
N1	16	-	19	-
N2	15	-	21	-
Unknown	0		5	
M status				
M0	53	-	64	-
M1	7	-	11	-
Unknown	0		5	
UICC TNM stage				
I	12	-	8	-
II	17	-	27	-
III	24	-	29	-
IV	7	-	11	-
Unknown	0		5	
Tumor differentiation				
Well	7	-	8	-
Moderate	40	-	53	-
Poor	13	-	12	-
Unknown	0		7	
Tumor size (mm)				
≥5	33	-	40	-
<5	27	-	35	-
Unknown	0		5	
Tumor location				
Right sided colon	16	-	22	-
Left sided colon	15	-	28	-
Rectum	29	-	30	-
CEA level				
Low (<5ng/ml)	28	60	42	80
High (≥5ng/ml)	29	0	34	0
Unknown	3	0	4	0
CA 19-9 level				
Low (<27U/ml)	39	57	54	68
High (≥27U/ml)	17	1	21	3
Unknown	4	2	5	9
CA125 level				
Low (<35U/ml)	51	58	61	77
High (≥35U/ml)	5	0	12	1
Unknown	4	2	7	2
CA-153 level				
Low (<25U/ml)	52	55	64	69
High (≥25U/ml)	0	1	4	0
Unknown	8	4	12	11
CA-724 level				
Low (<6.9U/ml)	38	58	42	79
High (≥6.9U/ml)	10	0	17	1
Unknown	12	2	21	0

Then, we depicted ROC curves to evaluate the performance of these miRNAs in discriminating CRC from HC. As shown in Table 2 and Figure 4A, miR-21 had the best discriminatory performance with an AUC of 0.708, while miR-145 had the worst performance with an AUC of 0.677.

**Table 2:** Diagnostic performances of the 5 dysregulated circulating miRNAs in the training and validation cohort

Study cohort	miRNAs	Fold change	AUC(95%CI)	P value	Cut-off	Sensitivity(95%CI)	Specificity(95%CI)
	miR-15b	2.550	0.684(0.587-0.780)	0.0005	0.527	70.00%(56.79%-81.16%)	65.00%(51.60%-76.87%)
	miR-17	1.646	0.680(0.580-0.780)	0.0007	0.971	85.00%(73.43%-92.90%)	45.00%(32.12%-58.39%)
Training	miR-21	3.135	0.708(0.616-0.799)	<0.0001	0.686	71.67%(58.56%-82.55%)	58.33%(44.88%-70.93%)
	miR-26b	3.434	0.702(0.608-0.795)	0.0001	0.530	61.67%(48.21%-73.93%)	70.00%(56.79%-81.16%)
	miR-145	2.145	0.677(0.581-0.773)	0.0008	0.840	68.33%(55.04%-79.74%)	53.33%(40.00%-66.33%)
	miR-15b	2.659	0.624(0.535-0.712)	0.0071	0.527	50.00%(38.61%-61.39%)	68.75%(57.41%-78.65%)
	miR-17	1.767	0.660(0.576-0.745)	0.0005	0.971	67.50%(56.11%-77.55%)	62.50%(50.96%-73.08%)
Validation	miR-21	2.802	0.649(0.563-0.735)	0.0011	0.686	71.25%(60.05%-80.82%)	52.50%(41.02%-63.79%)
	miR-26b	2.796	0.708(0.627-0.789)	<0.0001	0.530	72.50%(61.38%-81.90%)	56.25%(44.70%-67.32%)
	miR-145	2.488	0.629(0.542-0.716)	0.0047	0.840	62.50%(50.96%-73.08%)	61.25%(49.70%-71.94%)

Binary logistic regression analysis was conducted to combine the 5 dysregulated miRNAs with 5 commonly used tumor markers (CEA, CA19-9, CA-724, CA153, and CA125). As a result, two diagnostic models were constructed. One consists of only 5 miRNAs (miR-15b, miR-17, miR-21, miR-26b, and miR-145) and its calculating formula was as follows: logit(P) = 0.1559 × miR-15b - 0.2063 × miR-17 + 0.0712 × miR-21 + 0.2252 × miR-26b + 0.1311 × miR-145. The other consists of the above 5 miRNAs plus one tumor marker (CEA), and its calculating formula was as follows: logit(P) = 0.5640 × CEA + 0.1758 × miR-15b - 0.2653 × miR-17 + 0.0466 × miR-21 + 0.1871 × miR-26b + 0.2369 × miR-145. As shown in Figure 4B, the ROC curves demonstrated that the combination of 5 miRNAs plus CEA has a better discriminatory performance, with an AUC of 0.85, when compared with the 5 miRNAs (AUC: 0.681) or CEA alone (AUC: 0.793).

### Diagnostic model confirmation in the validation phase

**Figure 3:**
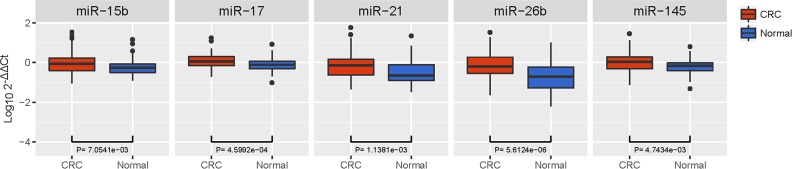
Box plot of the relative expression levels of dysregulated miRNAs in the validation cohort (n = 160) The y axis indicates log_10_ 2^−△△Ct^ relative expression of miRNA. All 5 dysregulated miRNAs exhibited similar expression patterns in the training phase (miR-15b, p = 0.0071; miR-17, p = 0.0005; miR-21, p = 0.0011; miR-26b, p < 0.0001; miR-145, p = 0.0047).

To confirm the discriminatory capability of the 5 dysregulated miRNAs and the diagnostic models detected in the training phase, an independent cohort of 80 CRC patients and 80 HC was utilized. As shown in Figure 3, we found by qRT-PCR analysis that all the dysregulated miRNAs exhibited similar expression patterns in the training phase (miR-15b, p = 0.0071; miR-17, p = 0.0005; miR-21, p = 0.0011; miR-26b, p < 0.0001; miR-145, p = 0.0047).

**Figure 4:**
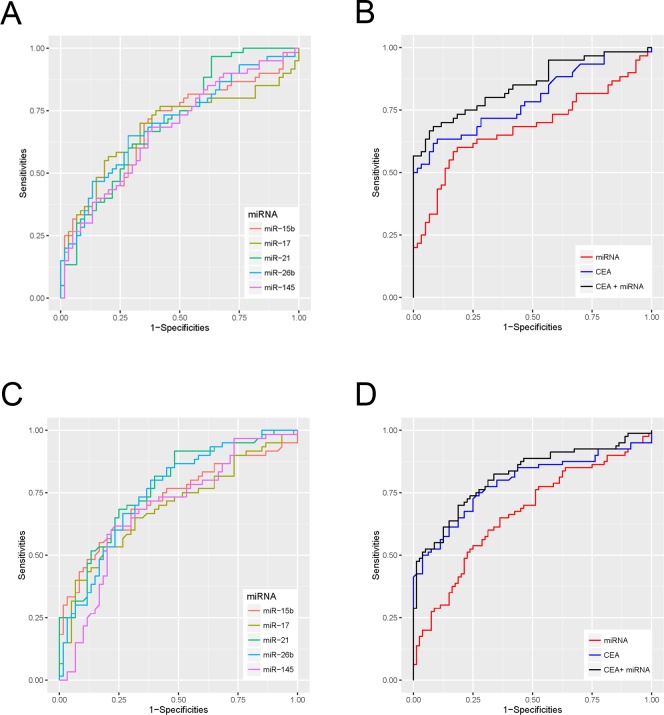
Receiver Operating Characteristic (ROC) curves for dysregulated miRNAs and diagnostic models in the training and the validation cohort **(A)** ROC curves for the diagnostic performances of dysregulated miRNAs in the training cohort. **(B)** ROC curves for the diagnostic performances of the 5 miRNAs + CEA, the 5 miRNAs alone, and CEA alone in the training cohort. **(C)** ROC curves for the diagnostic performance of the 5 dysregulated miRNAs in the validation cohort. **(D)** ROC curves for the diagnostic performances of 5 miRNAs + CEA, 5 miRNAs alone, and CEA alone in the validation cohort.

Roc curves were subsequently performed to validate the discriminatory performance of these miRNAs. MiR-26b had the best discriminatory performance (AUC: 0.708), while miR-15b had the worst performance (AUC: 0.624; Figure 4C and Table 2). Using the same formulas we further examined the efficiency of the diagnostic models constructed in the training cohort. As shown in Figure 4D, the model integrating 5 miRNAs and CEA had better discriminatory performance (AUC: 0.818) than the models comprising only the 5 miRNAs (AUC: 0.666) or just CEA (AUC: 0.790).

### Correlation between miRNAs and clinicopathological parameters in CRC

A summary of the correlations between the 5 dysregulated miRNAs identified above and the clinicopathological parameters of all the enrolled CRC patients (n = 140) is presented in Supplementary Table 2. MiR-26b expression was significantly correlated with cancer type (p = 0.018) and tumor size (p = 0.004), while miR-145 expression was significantly correlated with tumor size only (p = 0.047). No correlations were found between other circulating miRNA levels and clinicopathological parameters including age, gender, T status, N status, M status, TNM stage, tumor differentiation, tumor size, tumor location, cancer type, and CEA levels.

## DISCUSSION

Tumor-derived circulating miRNAs have attracted wide attention in the field of oncological diagnosis since Mitchell et al. first proposed that they could be stable biomarkers for blood-based detection of solid cancers [41, 42]. Although numerous studies have extensively investigated miRNAs as potential diagnostic markers for CRC, most of them failed to gain acceptance in clinical practice mainly due to insufficient validation, unstandardized methodologies, and lack of cross-validation in terms of population ethnicity [43, 44]. Therefore, identifying studies that can help improve diagnostic efficiency is of great clinical significance. In the present study, we performed a systemic literature review to select candidate circulating miRNAs previously identified in different study cohorts. We additionally enrolled 4 miRNAs found to be associated with malignant characteristics of CRC in our previous studies, thus defining a total of 30 candidate miRNAs for further screening.

In the training phase, we screened out 5 circulating miRNAs (miR-15b, miR-17, miR-21, miR-26b, and miR-145) that proved to be closely linked to the initiation and development of disease in CRC patients. For instance, miR-15b was suggested to promote the invasion and metastasis of CRC cells, while a protective effect was described instead for miR-145 [45, 46]. On the other hand, both miR-21 and miR-26b were identified as crucial drivers of colitis-associated carcinogenesis [9, 47], whereas miR-17 was shown to contribute to CRC progression by inducing epithelial-mesenchymal transition and cancer stem cell phenotype [48]. ROC analysis further demonstrated that miR-21 had the best performance in discriminating CRC patents from HC among these 5 miRNAs. This observation is consistent with several recent studies supporting circulating miR-21 as an effective CRC biomarker [39, 49, 50], although there are reports that question its practical utility in diagnosing CRC [51, 52]. As for miR-15b, miR-17, and miR-145, our results also agree with previous studies, and to our knowledge no related divergent reports were published [21, 38, 52]. Then, we employed a validation cohort to confirm the expression patterns of the 5 miRNAs selected in the training phase. Although these miRNAs remained significantly dysregulated in CRC patients, ROC analysis indicated that miR-26b has the best discriminating performance, which is somewhat inconsistent with our observation in the training phase. A recent work by Cristóbal et al. suggested that miR-26b overexpression might be correlated with lung metastasis in CRC [53]. Although to our knowledge there are currently no other available studies addressing circulating miR-26b levels in CRC patients, it should be mentioned that it had a stable and comparatively good discriminating performance both in the training and validation phases in our study, with an AUC of 0.702 and 0.708 respectively. Therefore, we conclude that miR-26b may be an effective plasma marker for CRC diagnosis.

Perhaps the most remarkable finding of our research was the construction and validation of a new diagnostic model integrating multiple miRNAs and CEA, a recognized CRC marker. Using logistic regression analysis, we found that our first diagnostic model, which combined the 5 screened miRNAs (miR-15b, miR-17, miR-21, miR-26b, and miR-145) had an inferior diagnostic performance as compared with the single most optimal miRNA (AUC in the training phase: 0.681 vs 0.708; AUC in the validation phase: 0.666 vs 0.708). This result is in accordance with a recent meta-analysis that suggested that a single circulating miRNA, miR-21, performed significantly better than various circulating miRNA panels in CRC diagnosis [54]. However, another meta-analysis proposed that multiple circulating miRNAs could dramatically improve diagnostic accuracy compared with individual ones, with AUC values ranging from 0.79 to 0.89 [55]. These divergent observations can be partly attributed to the heterogeneous expression of the several circulating miRNAs detected in CRC patients, and remain to be confirmed by further standardized clinical validations. We next compared the 5 miRNAs with CEA level and found that the miRNA panel had a significantly worse discriminating performance both in the training and validation phases. Although an opposite conclusion was reached by a previous study comparing multiple miRNAs with CEA level [20], our data thus suggests that the diagnostic efficiency of this miRNA combination set is inferior to current CEA detection. However, once integrated with CEA, our miRNA panel significantly improved the AUC for CEA, from 0.793 to 0.85 in the training phase, and from 0.790 to 0.818 in the validation phase. Although the validity of CEA determination in CRC diagnosis has been disputed, it remains the most commonly examined tumor marker for noninvasive diagnosis due to its high CRC specificity. The present results suggest that a novel model combining multiple circulating miRNAs and CEA level detection improves diagnostic efficiency in CRC, and could therefore be adopted successfully in the clinical practice. Furthermore, this approach appears to be more convenient and acceptable for patients in the diagnostic process, compared to conventional stool test and colonoscopy.

Despite its novel findings, our study has some potential deficiencies that are worth considering. Firstly, the majority of samples were obtained from stage III/IV patients, which prevented identifying eligible circulating miRNAs related to early stage CRC. This deficiency is largely attributed to the fact that most CRC patients are diagnosed at an advanced stage, while much fewer patients are diagnosed at stage I/II. To tackle this issue, more multicenter collaborations backed by appropriate clinical resources are strongly advocated. Secondly, in our study, combined miRNAs is inferior to single miR-21 or miR-26b in diagnostic performances, which is inconsistent with some studies. This issue is expected to be solved by further highly standardized validations in future. Thirdly, the diagnostic performance of our model is slightly inferior to that of other blood-based diagnostic markers such as methylated SPET9 (miRNAs + CEA, AUC: 0.85 (training)/0.818(validation) vs methylated SPET9, AUC: 0.88) [56]. In this regard, clinical validation is suggested in order to examine the efficacy and improve the construction of our model. On the other hand, additional efforts can be made to integrate miRNAs with other types of diagnostic biomarkers to create a more effective diagnostic or screening system. Finally, due to insufficient sampling we failed to identify dysregulated circulating miRNAs in patients with precancerous lesions such as advanced adenoma or ulcerative colitis. Therefore, their clinical utility in assessing CRC risk through population screening is worthy of further inquiry.

In conclusion, through systematic literature revision and clinical validation, our study identified 5 miRNAs differentially dysregulated in the plasma of CRC patients. By integrating these miRNAs and CEA level, we constructed and validated a new model that we believe will enhance the diagnostic accuracy of CRC and overcome some limitations of current blood-based diagnostic methods.

## MATERIALS AND METHODS

### Study design and miRNA selection

A flow chart of this study is illustrated in Figure 1. The study consisted of three general parts: a systematic literature review for selecting candidate miRNAs, a training phase for screening candidate miRNAs and constructing diagnostic models, and a validation phase for confirming optimal miRNAs/diagnostic models. For the literature review, we preliminarily selected candidate miRNAs from published studies based on the following inclusion criteria: diagnostic potential confirmed by at least 2 publications or CRC-related miRNAs identified in our previous studies. Then, we excluded unqualified candidates according to the following exclusion criteria: 1) miRNAs detected in tissue, feces, or whole blood; 2) obvious differences in methodology; 3) miRNAs detected in small samples (n ≤ 50). Next, the remaining candidates were screened in the training phase using qRT-PCR and integrated with traditional tumor markers (CEA, CA19-9, CA-724, CA153, and CA125) in diagnostic models. In the validation phase, the optimal diagnostic miRNAs and integrated models were examined in an independent cohort.

### Patient data and sample preparation

Between January 11, 2014 and April 8, 2016, a total of 60 newly diagnosed CRC patients and 60 healthy volunteers were enrolled from Shanghai Tenth People's Hospital Affiliated to Tongji University and allocated into a training cohort. In addition, a validation cohort including 80 newly diagnosed CRC patients and 80 healthy volunteers was enrolled from The Sixth People's Hospital Affiliated to Shanghai Jiao Tong University between July 6, 2011, and August 23, 2012. All the enrolled cases were clinicopathologically confirmed as CRC. None of the patients had family history of cancer nor received previous chemoradiotherapy treatment. Tumor Node Metastasis stage was classified according to the 7^th^ Union for International Cancer Control guidelines. The basic clinicopathological features of the two cohorts are presented in Table 1. This study was approved by the ethics committees of both hospitals and informed consent was obtained from all participants.

For sample preparation, 4 ml of peripheral blood was collected from each participant and transferred into EDTA tubes. Blood samples were centrifuged at 3000 rpm for 10 min and the supernatants were collected and stored at −80°C before further processing.

### RNA extraction and quantitative real-time PCR

Total RNA was extracted from plasma samples using an RNA isolation kit (Qiagen, Hilden, Germany) following the manufacturer's instructions. Briefly, 200 μl of plasma was thawed on ice, mixed with 1 ml QIAzol Lysis Reagent, and incubated at room temperature for 5 min. Inter-sample variation during RNA extraction was normalized using synthetic C. elegans cel-miR-39 (1.6 × 10^8^ copies/μl). Finally, the concentration of RNA samples was quantified on a NanoDrop ND-1000 spectrophotometer (Nano Drop Technologies, Wilmington, DE, USA).

The RNA thus obtained was reversely transcribed into cDNA using a MicroRNA Reverse Transcription Kit (BioTNT, Shanghai, China) according to the manufacturer's instructions. qRT-PCR was performed on a ViiA™ 7 Real-Time PCR System (Life Technologies, USA) under the following conditions: 95°C for 5 min, followed by 40 cycles at 95°C for 5 s, and 60° for 30 s. The reaction mixtures included 1 μl cDNA, 5 μl 2×qPCR Premix, 1 μl microRNA upper primer and lower primer, and 2 μl RNase-free water. The specificity and identity of the reaction products were verified by dissociation curve analysis. Data normalization was conducted using an exogenous (cel-miR-39) and an endogenous (miR-16-5p) control as described previously [57]. The relative level of each miRNA was calculated using the 2^−ΔΔCt^ method and all the assays were carried out in triplicate. Information of the primers used for miRNA amplification is supplied in Supplementary Table 3.

### Statistical analysis

Data are presented as mean ± SD. The baseline clinicopathological characteristics of each cohort were compared by student t test or chi-square test. Relative miRNAs levels in the CRC and HC groups were compared by Mann-Whitney test and two-sided α level was adjusted by simple Bonferroni correction. Correlations between miRNA levels and clinicopathological features were analyzed by Mann-Whitney tests. ROC curves and AUC values were used to describe the diagnostic performance of miRNAs and diagnostic models. The diagnostic models were constructed using binary logistic regression analysis. In brief, an accessing language procedure was used to integrate the miRNAs and serum tumor markers. A scoring formula was then established by assigning the coefficient to each included variable and the score of each patient was calculated accordingly. All the statistical analyses were performed using R version 3.2.3. A p < 0.05 was considered statistically significant.

## SUPPLEMENTARY TABLES








