# Supplementary material for: Systematic literature review and clinical validation of circulating microRNAs as diagnostic biomarkers for colorectal cancer
Source: Oncotarget. 2017 Jul 18;8(40):68317–28. doi: 10.18632/oncotarget.19344 (PMC5620259; doi:10.18632/oncotarget.19344)
Supplement: Supplementary file 1 [file oncotarget-08-68317-s001.pdf]

Systematic literature review and clinical validation of circulating microRNAs as diagnostic biomarkers for colorectal cancer

SUPPLEMENTARY MATERIALS

Supplementary Table 1: Characteristics of 26 CRC-related circulating miRNAs in reported studies.

See Supplementary File 1

Supplementary Table 2: Correlations between the levels of dysregulated miRNAs and the clinicopathological characteristics of colorectal cancer patients.

See Supplementary File 2

Supplementary Table 3: Sequences of the primers used for miRNAs in the study.

See Supplementary File 3
