# Supplementary material for: Systematic literature review and clinical validation of circulating microRNAs as diagnostic biomarkers for colorectal cancer
Source: Oncotarget. 2017 Jul 18;8(40):68317–28. doi: 10.18632/oncotarget.19344 (PMC5620259; doi:10.18632/oncotarget.19344)
Supplement: Supplementary file 2 [file oncotarget-08-68317-s002.doc]

**Supplementary Table 1**: Characteristics of 26 CRC-related circulating miRNAs in reported studies

| miRNAs | Year | References | Country | Expression | P value | CRC | HC | Median  Age | Sources | Normalization | AUC | Cut-off | Sen | Spe |
| --- | --- | --- | --- | --- | --- | --- | --- | --- | --- | --- | --- | --- | --- | --- |
| miR-15b | 2013 | Giraldez[21] | Spain | ↑ | 0.002 | 53 | 82 | 63 | plasma | miR-16 | NA | NA | NA | NA |
|  | 2013 | Kanaan[22] | America | ↑ | 0.0009 | 45 | 26 | NA | plasma | RNU6B | NA | NA | NA | NA |
| miR-17-3p | 2009 | Ng[12] | China | ↑ | <0.0001 | 90 | 50 | 71 | plasma | RNU6B | 0.717 | 3.6 | 64 | 70 |
|  | 2012 | Faltejskova[23] | Czech | - | 0.18 | 100 | 30 | 67 | serum | miR-16 | - | - | - | - |
| miR-18a | 2013 | Luo[38] | Germany | ↑ | <0.0001 | 80 | 144 | 68 | plasma | miR-16 | 0.5653 | 8.383 | NA | NA |
|  | 2013 | Brunet[24] | Spain | ↑ | <0.05 | 30 | 26 | 64 | serum | miR-16/let7a /miR-103 | NA | NA | NA | NA |
|  | 2013 | Giraldez[21] | Spain | ↑ | <0.001 | 53 | 82 | 63 | plasma | miR-16 | NA | NA | NA | NA |
|  | 2013 | Liu [14] | China | - | 0.093 | 200 | 80 | 51 | serum | miR-16 | - | - | - | - |
| miR-19a | 2013 | Giraldez[21] | Spain | ↑ | <0.001 | 53 | 82 | 63 | plasma | miR-16 | NA | NA | NA | NA |
| miR-19b | 2013 | Giraldez[21] | Spain | ↑ | <0.001 | 53 | 82 | 63 | plasma | miR-16 | NA | NA | NA | NA |
| miR-21 | 2010 | Pu[15] | China | - | NA | 103 | 37 | 58 | plasma | NA | - | - | - | - |
|  | 2012 | Wang[25] | China | ↑ | <0.001 | 32 | 39 | 63 | plasma | miR-16 | 0.85 | 3.59 | 87.5 | 74.4 |
|  | 2012 | Kanaan[13] | America | ↑ | NA | 20 | 20 | 57 | plasma | RNU6B | 0.91 | NA | 90 | 90 |
|  | 2013 | Liu [14] | China | ↑ | <0.001 | 200 | 80 | 51 | plasma | miR-16 | 0.802 | 0.0043 | 65 | 85 |
|  | 2013 | Luo[38] | Germany | ↑ | <0.0001 | 80 | 144 | 68 | plasma | miR-16 | 0.6528 | 3.703 | 51.66 | 80.72 |
|  | 2013 | Toiyama[18] | Japan | ↑ | <0.001 | 186 | 53 | 67 | serum | cel-miR-39 | 0.927 | 0.0019 | 82.8 | 90.6 |
|  | 2013 | Brunet[24] | Spain | - | 0.087 | 30 | 26 | 64 | serum | miR-16/let7a /miR-103 | - | - | - | - |
|  | 2014 | Nonaka[26] | Japan | ↑ | 0.003 | 84 | 32 | NA | serum | cel-miR-39/  RNU6B | 0.675 | 0.0107 | 54.7 | 84.4 |
|  | 2014 | Basati[27] | Iran | ↑ | 0.0001 | 40 | 40 | 55 | serum | RNU6B | 0.87 | 1.49 | 77 | 78 |
|  | 2014 | Zanutto[28] | Italy | ↑ | 0.046 | 29 | 29 | NA | plasma | miR-16 | 0.647 | NA | NA | NA |
|  | 2014 | Du[29] | China | ↑ | <0.001 | 49 | 49 | NA | plasma | cel-miR-39 | 0.877 | 0.0022 | 76.2 | 93.2 |
| miR-24 | 2015 | Fang[19] | China | ↓ | <0.05 | 111 | 130 | 60 | plasma | cel-miR-39 | 0.839 | -1.731 | 78.38 | 83.85 |
| miR-29a | 2010 | Huang[30] | China | ↑ | <0.001 | 100 | 59 | 61 | plasma | miR-16 | 0.769 | 1.33 | 69 | 89.1 |
|  | 2012 | Faltejskova[23] | Czech | - | 0.14 | 100 | 30 | 67 | serum | miR-16 | - | - | - | - |
|  | 2013 | Brunet[24] | Spain | ↑ | <0.05 | 30 | 26 | 64 | serum | miR-16/let7a /miR-103 | NA | NA | NA | NA |
|  | 2013 | Luo[38] | Germany | ↑ | 0.001 | 80 | 144 | 68 | plasma | miR-16 | 0.5714 | 7.2 | 30.46 | 90.36 |
|  | 2013 | Hofsli[31] | Norway | ↑ | NA | 40 | 10 | 70 | serum | NA | NA | NA | NA | NA |
|  | 2013 | Giraldez[21] | Spain | ↑ | <0.003 | 53 | 82 | 63 | plasma | miR-16 | NA | NA | NA | NA |
|  | 2014 | Zanutto[28] | Italy | - | NA | 36 | 42 | NA | plasma | miR-16 | - | - | - | - |
| miR-34a | 2013 | Brunet[24] | Spain | - | 0.055 | 30 | 26 | 64 | serum | miR-16/let7a /miR-103 | - | - | - | - |
|  | 2015 | Aherne[32] | Ireland | ↑ | <0.001 | 40 | 40 | NA | Plasma | let-7e | NA | NA | NA | NA |
| miR-92a | 2009 | Ng[12] | China | ↑ | <0.001 | 90 | 50 | 71 | plasma | RNU6B | 0.885 | 240 | 89 | 70 |
|  | 2010 | Huang[30] | China | ↑ | <0.001 | 100 | 59 | 61 | plasma | miR-16 | 0.838 | 1.231 | 84 | 71.2 |
|  | 2012 | Faltejskova[23] | Czech | - | 0.6 | 100 | 30 | 67 | serum | miR-16 | - | - | - | - |
|  | 2013 | Liu [14] | China | ↑ | <0.001 | 200 | 80 | 51 | serum | miR-16 | 0.847 | 0.00017 | 65.5 | 82.5 |
|  | 2013 | Giraldez[21] | Spain | ↑ | 0.003 | 53 | 82 | 63 | plasma | miR-16 | NA | NA | NA | NA |
|  | 2013 | Luo[38] | Germany | ↑ | 0.004 | 80 | 144 | 68 | plasma | miR-16 | 0.5609 | 2.87 | 68.21 | 46.4 |
|  | 2013 | Hofsli[31] | Norway | ↑ | NA | 40 | 10 | 70 | serum | NA | NA | NA | NA | NA |
|  | 2013 | Brunet[24] | Spain | - | 0.087 | 30 | 26 | 64 | serum | miR-16/let7a /miR-103 | - | - | - | - |
|  | 2014 | Du[29] | China | - | 0.443 | 49 | 49 | NA | plasma | cel-miR-39 | - | - | - | - |
| miR-125b | 2015 | Yamada[39] | Japan | ↑ | NA | 12 | 77 | NA | serum | cel-miR-39 | NA | NA | NA | NA |
| miR-139-3p | 2013 | Kanaan[22] | America | NA | 0.0009 | 45 | 26 | NA | plasma | RNU6B | NA | NA | NA | NA |
| miR-145 | 2013 | Luo[38] | Germany | ↑ | 0.0004 | 80 | 144 | 68 | plasma | miR-16 | 0.5841 | 9.26 | 43.05 | 74.7 |
| miR-183 | 2014 | Yuan[33] | China | ↑ | <0.0001 | 118 | 61 | NA | plasma | RNU6B | 0.829 | NA | 73.7 | 88.5 |
| miR-194 | 2015 | Basati[34] | Iran | ↓ | <0.0001 | 55 | 55 | 58.5 | serum | cel-miR-39 | 0.85 | 1.08 | 72 | 80 |
| miR-199a-3p | 2013 | Hofsli[31] | Norway | ↓ | <0.01 | 40 | 10 | 70 | serum | NA | NA | NA | NA | NA |
|  | 2014 | Nonaka[26] | Japan | ↑ | 0.016 | 84 | 32 | NA | serum | cel-miR-39/  RNU6B | 0.644 | 0.0010 | 47.6 | 75.0 |
| miR-221 | 2010 | Pu[15] | China | ↑ | 0.0021 | 103 | 37 | 58 | plasma | NA | 0.606 | 1.69 | 86 | 41 |
|  | 2013 | Hofsli[31] | Norway | ↓ | <0.01 | 40 | 10 | 70 | serum | NA | NA | NA | NA | NA |
| miR-320a | 2013 | Hofsli[31] | Norway | ↑ | <0.01 | 40 | 10 | 70 | serum | NA | NA | NA | NA | NA |
|  | 2015 | Fang[19] | China | ↓ | <0.01 | 111 | 130 | 60 | plasma | cel-miR-39 | 0.886 | -1.006 | 92.79 | 73.08 |
| miR-331-3p | 2013 | Kanaan[22] | America | NA | 0.0011 | 45 | 26 | NA | plasma | RNU6B | NA | NA | NA | NA |
| miR-372 | 2015 | Yu[35] | China | ↑ | ＜0.001 | 165 | 30 | 60.7 | serum | NA | 0.854 | NA | 81.9 | 73.3 |
| miR-378 | 2013 | Hofsli[31] | Norway | ↑ | <0.01 | 40 | 10 | 70 | serum | NA | NA | NA | NA | NA |
|  | 2014 | Zanutto[28] | Italy | ↑ | 0.001 | 29 | 29 | NA | plasma | miR-16 | 0.796 | NA | NA | NA |
| miR-423 | 2015 | Fang[19] | China | ↓ | <0.001 | 111 | 130 | 60 | plasma | cel-miR-39 | 0.833 | -0.854 | 91.89 | 70.77 |
|  | 2013 | Hofsli[31] | Norway | ↑ | NA | 40 | 10 | 70 | serum | NA | NA | NA | NA | NA |
| miR-431 | 2013 | Kanaan[22] | America | NA | 0.0008 | 45 | 26 | NA | plasma | RNU6B | NA | NA | NA | NA |
| miR-592 | 2015 | Liu[36] | China | ↑ | <0.05 | 62 | 98 | NA | serum | RNU6B | NA | NA | NA | NA |
| miR-601 | 2012 | Wang[37] | China | ↓ | <0.0001 | 90 | 58 | 60 | plasma | cel-miR-39/  miR-16 | 0.747 | -11.5 | 69.2 | 72.4 |
| miR-760 | 2012 | Wang[37] | China | ↓ | <0.0001 | 90 | 58 | 60 | plasma | cel-miR-39/  miR-16 | 0.788 | -8.09 | 80 | 72.4 |

CRC: colorectal cancer; HC: healthy controls; NA: not applicable; “–“”: no significance; Sen: sensitivity; Spe: specificity
