# Supplementary material for: Systematic literature review and clinical validation of circulating microRNAs as diagnostic biomarkers for colorectal cancer
Source: Oncotarget. 2017 Jul 18;8(40):68317–28. doi: 10.18632/oncotarget.19344 (PMC5620259; doi:10.18632/oncotarget.19344)
Supplement: Supplementary file 3 [file oncotarget-08-68317-s003.docx]

**Supplementary Table 2: Correlations between the levels of dysregulated miRNAs and the clinicopathological characteristics of colorectal cancer patients**

| Characteristics | No. of patients | miR-15b | P value | miR-17 | P value | miR-21 | P value | miR-26b | P value | miR-145 | P value |
| --- | --- | --- | --- | --- | --- | --- | --- | --- | --- | --- | --- |
| Age |  |  |  |  |  |  |  |  |  |  |  |
| ≥65 year | 65 | 2.61±3.99 | 0.802 | 1.71±2.18 | 0.748 | 2.24±3.98 | 0.340 | 3.38±7.02 | 0.162 | 2.41±4.70 | 0.519 |
| <65 year | 75 | 2.58±4.74 |  | 1.77±3.04 |  | 3.73±9.49 |  | 2.41±6.05 |  | 2.36±5.10 |  |
| Gender |  |  |  |  |  |  |  |  |  |  |  |
| Female | 54 | 2.94±4.28 | 0.283 | 1.98±3.23 | 0.281 | 2.09±2.88 | 0.423 | 3.07±5.00 | 0.125 | 3.21±5.73 | 0.060 |
| male | 86 | 2.38±4.39 |  | 1.58±2.13 |  | 3.46±8.75 |  | 2.84±7.43 |  | 1.87±4.20 |  |
| T status |  |  |  |  |  |  |  |  |  |  |  |
| T1/2 | 21 | 2.66±4.47 | 0.666 | 1.80±2.85 | 0.969 | 3.33±7.78 | 0.172 | 2.71±5.59 | 0.678 | 2.17±4.24 | 0.796 |
| T3/4 | 114 | 2.69±4.17 |  | 1.31±0.98 |  | 1.37±2.01 |  | 3.51±9.82 |  | 3.66±7.76 |  |
| N status |  |  |  |  |  |  |  |  |  |  |  |
| Present | 71 | 2.32±3.57 | 0.900 | 1.56±2.20 | 0.537 | 2.52±5.60 | 0.594 | 2.36±4.28 | 0.504 | 2.78±6.31 | 0.667 |
| Absent | 64 | 2.95±5.17 |  | 1.87±3.07 |  | 3.54±8.69 |  | 3.43±8.13 |  | 2.47±5.03 |  |
| M status |  |  |  |  |  |  |  |  |  |  |  |
| Present | 18 | 2.27±3.94 | 0.497 | 2.71±3.88 | 0.308 | 2.40±3.97 | 0.683 | 1.92±3.34 | 0.153 | 1.43±1.75 | 0.216 |
| Absent | 117 | 2.73±4.49 |  | 1.57±2.39 |  | 3.12±7.60 |  | 2.97±6.73 |  | 2.55±5.26 |  |
| TNM stage |  |  |  |  |  |  |  |  |  |  |  |
| I/II | 64 | 3.06±5.18 | 0.937 | 1.89±3.08 | 0.565 | 3.57±8.68 | 0.574 | 3.45±8.13 | 0.428 | 2.52±5.03 | 0.526 |
| III/IV | 71 | 2.32±3.57 |  | 1.58±2.21 |  | 2.53±5.60 |  | 2.28±4.24 |  | 2.30±4.90 |  |
| Differentiation |  |  |  |  |  |  |  |  |  |  |  |
| Poor | 25 | 3.53±5.10 | 0.879 | 1.57±1.33 | 0.626 | 2.42±3.17 | 0.508 | 3.81±8.93 | 0.095 | 3.93±7.54 | 0.201 |
| Well/moderate | 108 | 2.51±4.26 |  | 1.76±2.90 |  | 3.20±7.93 |  | 2.62±5.74 |  | 2.07±4.15 |  |
| Tumor size |  |  |  |  |  |  |  |  |  |  |  |
| ≥50mm | 73 | 2.23±4.34 | 0.499 | 1.50±2.23 | 0.064 | 2.03±3.77 | 0.952 | 1.41±2.18 | **0.004** | 2.09±4.77 | **0.047** |
| <50mm | 62 | 3.19±4.46 |  | 1.99±3.07 |  | 4.20±9.75 |  | 4.51±8.88 |  | 2.77±5.16 |  |
| Tumor location |  |  |  |  |  |  |  |  |  |  |  |
| Left sided | 43 | 2.63±5.54 | 0.413 | 1.90±3.67 | 0.441 | 2.95±6.95 | 0.561 | 2.45±7.19 | 0.222 | 2.67±5.99 | 0.817 |
| Right sided | 38 | 1.95±3.43 |  | 1.55±1.28 |  | 2.09±3.85 |  | 2.11±4.36 |  | 1.53±1.93 |  |
| Cancer type |  |  |  |  |  |  |  |  |  |  |  |
| Colon | 81 | 2.31±4.66 | 0.070 | 1.74±2.80 | 0.652 | 2.55±5.69 | 0.349 | 2.29±6.00 | **0.018** | 2.14±4.57 | 0.273 |
| Rectal | 59 | 2.99±3.87 |  | 1.73±2.33 |  | 3.46±8.70 |  | 3.81±7.27 |  | 2.73±5.28 |  |
| CEA level |  |  |  |  |  |  |  |  |  |  |  |
| ≥5ng/mL | 63 | 3.18±4.60 | 0.185 | 2.03±3.59 | 0.960 | 4.17±9.45 | 0.091 | 3.19±6.46 | 0.147 | 2.42±5.10 | 0.657 |
| <5ng/mL | 70 | 2.27±4.26 |  | 1.45±1.36 |  | 2.06±4.31 |  | 2.30±4.72 |  | 2.45±4.91 |  |
