# Supplementary material for: Systematic literature review and clinical validation of circulating microRNAs as diagnostic biomarkers for colorectal cancer
Source: Oncotarget. 2017 Jul 18;8(40):68317–28. doi: 10.18632/oncotarget.19344 (PMC5620259; doi:10.18632/oncotarget.19344)
Supplement: Supplementary file 4 [file oncotarget-08-68317-s004.docx]

| **miRNA** | **Accession** | **Forward 5’-3’** | **Reverse 5’-3’** |
| --- | --- | --- | --- |
| hsa-miR-15b | MIMAT0000417 | GCAGCACATCATGGTTTAC | GGTCCACCATACCACTCCA |
| hsa-miR-17-3p | MIMAT0000071 | GCAGTGAAGGCACTTGT | CACCATACCACTCCAGAT |
| hsa-miR-17 | MIMAT0000070 | AAAGTGCTTACAGTGCAG | CACCATACCACTCCAGAT |
| hsa-miR-18a | MIMAT0000072 | AAGGTGCATCTAGTGCAG | GGTCCACCATACCACTC |
| hsa-miR-19a | MIMAT0000073 | GCAAATCTATGCAAAACTG | GGTCCACCATACCACTC |
| hsa-miR-19b | MIMAT0000074 | GCAAATCCATGCAAAACTG | GGTCCACCATACCACTCCA |
| hsa-miR-21 | MIMAT0000530 | AGCTTATCAGACTGATGTTG | CACCATACCACTCCAGAT |
| hsa-miR-24 | MIMAT0000080 | GGCTCAGTTCAGCAGG | CACCATACCACTCCAGAT |
| hsa-miR-26b | MIMAT0000083 | TTCAAGTAATTCAGGATAGG | CACCATACCACTCCAGAT |
| hsa-miR-29a | MIMAT0000086 | TAGCACCATCTGAAATCG | CACCATACCACTCCAGAT |
| hsa-miR-34a | MIMAT0000255 | GCAGTGTCTTAGCTGGTT | CACCATACCACTCCAGAT |
| hsa-miR-92a | MIMAT0000092 | CCTATTGCACTTGTCCC | CACCATACCACTCCAGAT |
| hsa-miR-125b | MIMAT0000423 | TCCCTGAGACCCTAACTT | CACCATACCACTCCAGAT |
| hsa-miR-139-3p | MIMAT0004552 | GGCCCTGTTGGAGTAA | GGTCCACCATACCACTC |
| hsa-miR-145 | MIMAT0000437 | GTCCAGTTTTCCCAGG | CACCATACCACTCCAGAT |
| hsa-miR-149 | MIMAT0000450 | CTGGCTCCGTGTCTTCACT | CACCATACCACTCCAGAT |
| hsa-miR-150 | MIMAT0000451 | TCTCCCAACCCTTGTACC | CACCATACCACTCCAGAT |
| hsa-miR-183 | MIMAT0000212 | GCTATGGCACTGGTAGAA | GGTCCACCATACCACTC |
| hsa-miR-194 | MIMAT0000460 | GTAACAGCAACTCCATGTG | CACCATACCACTCCAGAT |
| hsa-miR-199a-3p | MIMAT0000232 | GTAGTCTGCACATTGGT | CACCATACCACTCCAGAT |
| hsa-miR-221* | MIMAT0004568 | ACCTGGCATACAATGTAGAT | CACCATACCACTCCAGAT |
| hsa-miR-320a | MIMAT0000510 | AAAAGCTGGGTTGAGA | CACCATACCACTCCAGAT |
| hsa-miR-331-3p | MIMAT0000760 | CCTGGGCCTATCCTAGA | GTCCACCATACCACTCCA |
| hsa-miR-372 | MIMAT0000724 | AAGTGCTGCGACATTTG | CACCATACCACTCCAGAT |
| hsa-miR-378* | MIMAT0000742 | CTGACTCCAGGTCCTGT | CACCATACCACTCCAGAT |
| hsa-miR-423 | MIMAT0004748 | GCAGAGAGCGAGACTT | GGTCCACCATACCACTC |
| hsa-miR-431 | MIMAT0001625 | GTCTTGCAGGCCGTCAT | GGTCCACCATACCACTCCA |
| hsa-miR-592 | MIMAT0003260 | GTGTCAATATGCGATGATGT | CACCATACCACTCCAGAT |
| hsa-miR-760 | MIMAT0004957 | CGGCTCTGGGTCTGT | CACCATACCACTCCAGAT |
| hsa-miR-601 | MIMAT0003269 | GTCTAGGATTGTTGGAGG | GGTCCACCATACCACTC |
| hsa-miR-16 | MIMAT0000069 | AGCAGCACGTAAATATTGG | CACCATACCACTCCAGAT |
| cel-miR-39-3p | MIMAT0000010 | TCACCGGGTGTAAATCAG | TCCACCATACCACTCCAG |

**Supplementary Table 3: Sequences of the primers used for miRNAs in the study**
